# Alcohol and Cardiovascular Diseases—Do the Consumption Pattern and Dose Make the Difference?

**DOI:** 10.3390/jcdd9100317

**Published:** 2022-09-22

**Authors:** Małgorzata Chudzińska, Łukasz Wołowiec, Joanna Banach, Daniel Rogowicz, Grzegorz Grześk

**Affiliations:** 1 Department of Nutrition and Dietetics, Ludwik Rydygier Collegium Medicum in Bydgoszcz, Nicolaus Copernicus University in Toruń, Dębowa 3 Street, 85-626 Bydgoszcz, Poland; 2 Department of Cardiology and Clinical Pharmacology, Ludwik Rydygier Collegium Medicum in Bydgoszcz, Nicolaus Copernicus University in Toruń, Ujejskiego 75 Street, 85-168 Bydgoszcz, Poland

**Keywords:** alcohol, wine, cardiovascular diseases, light and moderate drinking, compulsive and binge drinking, atrial fibrillation

## Abstract

Excessive consumption of alcohol is not only a social problem, but it also significantly increases the morbidity and mortality rates of many societies. A correlation has been demonstrated between alcohol consumption and increased mortality from cancer, accidents and injuries, liver cirrhosis and other causes. Alcohol abuse increases the incidence of hemorrhagic stroke and the risk of ischemic stroke, induces serious arrhythmias, adversely affects blood pressure and damages the heart muscle. The dose and way of drinking alcohol play a crucial role in assessing whether this drink allows people to maintain health or whether it is a great health and social threat. The beneficial effects of low and moderate doses of alcohol on the occurrence of cardiovascular diseases have been shown in many population studies and meta-analyses in which the effect of U-shaped or J-shaped curves relating alcohol intake to cardiovascular mortality was observed, especially in ischemic heart disease. However, due to the fact that alcohol consumption is associated with many health hazards, it is not recommended to consume it as a preventive action of cardiovascular diseases. Moreover, recent studies suggest that association of low-to-moderate alcohol consumption with the reduction in cardiovascular risk is a result of lifestyle changes and that any reduction in alcohol consumption is in fact beneficial in terms of general health.

## 1. Introduction

Alcohol is one of the most important risk factors for mortality [1]. Recent studies conducted in 189 countries for the purposes of the Global Burden of Disease project show that the three main factors of health loss (the so-called DALY indicator—the disability-adjusted life years) are, respectively: hypertension (7%), smoking (6%) and alcohol drinking (5.5%) [2]. Alcohol consumption is a causal factor in more than 200 diseases (especially non-infectious diseases) and injuries [3]. The risk for disease development increases in proportion to the dose of alcoholic beverage consumed. On a global scale, global alcohol intake is showing a steady upward trend—from 5.9 L of pure alcohol per year per adult in 1990 to 6.5 L in 2017, and it is expected to increase to 7.6 L by 2030 [4]. Estimates suggest that by 2030, half of adults will drink alcohol on a regular basis and almost a quarter (23%) will drink at least once a month. In most regions of the world, especially in low-income countries, the amount of alcohol consumed is currently growing faster than the number of drinkers, which not only increases the percentage of heavy drinking episodes, but also inevitably leads to an increasing burden of alcohol-related diseases [4]. Although Mendelian randomized, controlled trials show that abstainers have the lowest risk of cardiovascular events and that any dose of alcohol may be harmful, the above data show that alcohol has deeply embedded in the lifestyle of many societies and there is no indication that it will change even in the distant future [5,6]. If this phenomenon has to be accepted, then it may be worthwhile to have a closer look at the results of many clinical and observational studies on what dose and pattern of alcohol consumption are most harmful to health and emphasize that the types of alcohol are unequal in terms of their cardiovascular effects.

## 2. Wine in Moderation—The Joy of the Soul and Heart

This sentence expressed in the biblical Book of Sirach is still valid in spite of the passage of several thousand years [7]. In 1926, American biologist Raymond Perl showed that people who drink moderate amounts of alcohol have a longer lifespan than both non-drinkers and heavy drinkers. He was the first researcher to observe the U-shaped relationship between mortality and the amount of alcohol consumed [8].

The published results of a 13-year prospective observational study involving a group of 12,000 British physicians clearly indicated the beneficial effects of moderate alcohol consumption on the cardiovascular system [9]. These observations were confirmed by a 7-year observation of the German population that participated in the German National Health Surveys. The lowest risk of death from cardiovascular causes and death from all causes was observed in the group of men consuming up to 20 g of alcohol per day. Compared with abstainers, the risk was lower by nearly 50% [10].

Part of the observational Seven Countries study of the Italian population living in rural regions also showed a link between moderate, regular alcohol consumption and life expectancy [6]. In the group of men aged 45–64 consuming up to 60 g of alcohol per day, mainly wine, the expected life expectancy from the time of inclusion in the study was over 21 years and was higher by an average of 2 years, compared to abstainers and alcohol abusers (>10 “drinks” per day). In men (1536 Italian males, aged 30–69, 30 years—1096 deaths), the model assessing the number of deaths related to coronary heart disease, which were prevented by moderate consumption of wine and the number of coronary deaths caused by consumption of excessive amounts of ethanol. This model showed that low-to-moderate amounts of alcohol prevented about 400 deaths due to coronary heart disease each year, which resulted in a 14% reduction in mortality. During the same period, 20 coronary deaths were associated with alcohol abuse [11].

In the majority of observational prospective studies and meta-analyses, it was shown that the relationship between alcohol consumption and the incidence of coronary artery disease and stroke took the shape of the letters U or J on the graph. The lowest risk of cardiovascular disease and coronary artery disease was observed in people consuming moderate amounts of alcohol. This risk increased especially in people abusing alcohol, but also in non-drinkers. There was a higher risk of falling ill and dying, mainly due to injuries, poisoning, suicide, liver cirrhosis, cancer and stroke observed in heavy drinkers [12,13,14,15]. This relationship was well-illustrated by the results of the American Cancer Prevention Study II, which evaluated drinking habits and other lifestyle-related factors in a group of nearly half a million American adults [12]. At the same time, the causes of deaths in this group were analyzed during the next nine years of observation. The lowest risk of death was observed in persons of both sexes who consumed 1–2 portions of alcohol daily [10]. In the meta-analysis by Ronksley et al., the authors showed an increased risk of death and a higher incidence of stroke with daily alcohol consumption in the range of 50–60 g and more, whereas lower doses of alcohol had a beneficial effect in virtually all studies analyzed [14].

Through the analyses of data from nine representative surveys from the districts keeping the National Health Interview Survey (NHIS) in the United States in 1987–2000 and including data of 245,207 people, it was found that low and moderate alcohol consumption (slightly more than one drink daily) may be a factor that reduces the risk of cardiovascular death. Among those who were lifelong abstainers or had been rare-drinkers in the past and those who stopped drinking, the risk of dying from cardiovascular disease was higher than in moderate drinkers [16].

One of the effects of alcohol consumed in low doses may be also a reduced risk of sudden cardiac death. The US Physicians Health Study, which evaluated patients without diagnosed coronary artery disease, showed that the relative risk of sudden cardiac death was 0.4 and 0.2 in men consuming 2–4 or 5–6 portions of alcohol per week compared to non-drinkers; this risk was higher and approached unity when consuming two portions of alcohol a day. A typical U-shaped curve of incident dependence on alcohol consumption was observed here [17]. The mechanism of alcohol action in sudden cardiac death remains incompletely understood, but it is thought to be associated with a reduction in the risk of ventricular arrhythmia induced by acute coronary episodes [18]. In spite of the accepted knowledge that ethanol acutely modulates numerous targets in cardiomyocytes, including ion channels, Ca^2+^-handling proteins and gap junctions, the actual impact of ethanol on cardiac electrophysiology and reentrant arrhythmias is still unclear. Sutanto et al. postulate ethanol concentration-dependent electrophysiologic phenomenon eliciting both the inducibility and stability of reentrant arrhythmias, particularly in the presence of disease-associated remodeling. Another interesting observation was an anti-fibrillatory effect of low levels of ethanol within atrial muscle [19].

It is difficult to estimate the level of alcohol consumption that would be associated with the strongest protective effect against coronary artery disease, as different measures and definitions of alcohol consumption were used in different studies. According to Mukamal et al., about 30 g of ethanol consumed on a regular basis can contribute to reducing the risk of death, coronary heart disease or stroke [16]. A meta-analysis of 51 studies published by Corrao et al. showed that the risk of coronary heart disease was the lowest when consuming 20–25 g of ethanol per day [15].

Most researchers claim that the strongest protective effect is associated with the consumption of 20–30 g of alcohol per day, which corresponds to 2–3 portions of alcoholic beverages, the so-called drinks. Usually one unit of alcohol (10 g) is taken as 240–300 mL 5% alcohol by volume of beer, 100–125 mL 12% alcohol by volume wine or 30–37.5 mL 40% alcohol by volume spirits. Heavy drinking is defined as a long-term, high-dose intake of >60 g/day in men and >40 g/day in women [20,21]. ”Standard drink” varies considerably in different countries. For example, in the UK, one standard unit equals 8 g of pure alcohol, in the USA it amounts to 14 g per standard drink and in Canada it is 3.6 g [22,23].

Based on a meta-analysis of 42 studies, Rimm et al. showed a beneficial relationship between the consumption of 30 g of ethanol and changes in high-density lipoprotein (HDL) cholesterol, apolipoprotein I, triglycerides and selected hemostatic risk factors that can reduce the risk of coronary heart disease by nearly 25% [24]. The beneficial effects of moderate doses of alcohol on the cardiovascular system in people with a cardiovascular history were confirmed by the results of a large meta-analysis from 54 publications by Costanzo et al. Eight prospective studies on patients with a history of cardiovascular diseases (CVD) were selected from them. Alcohol was shown to be protective in patients with documented cardiovascular disease, as alcohol consumption at low and moderate doses (5–26 g/day) was clearly associated with a reduction in both general and cardiovascular mortality. The curve of this relationship was shaped like the letter J [25]. The same authors in the meta-analysis of 16 prospective studies on the relationship between wine consumption and the occurrence of cardiovascular events reported the most beneficial effect for the dose of 21 g ethanol/d, which remained up to 72 g/d. The risk of death from cardiovascular causes was the lowest with a daily consumption of wine containing 24 g of ethanol, and the reduction in the risk of death from other causes was the highest for 10 g/d. The analysis of spirit drinks did not confirm the existence of a beneficial relationship [26].

On the contrary, Wood et al. analyzed the individual-participant data from 599,912 current drinkers without previous cardiovascular diseases from three large-scale data sources in 19 high-income countries. They found that the threshold for lowest risk of all-cause mortality was about 100 g of alcohol/week. For cardiovascular diseases other than myocardial infarction (MI), there were no clear risk thresholds below which lower alcohol consumption stopped being associated with lower disease risk. This analysis shows the lower limits for alcohol consumption than those which are recommended in most current guidelines [27].

Although the interconnection between alcohol use and cardiovascular disease is affected by several behavioral, genetic and biological factors, the dose and frequency of alcohol consumption seem to have the greatest influence. Nevertheless, any positive aspects of low-to-moderate drinking must be balanced against significant physiological consequences [28].

Optimal, light/moderate alcohol consumption means that you need to remain at the lowest point of the J-shaped curve for alcohol use in order to take advantage of potential cardioprotection, which may appear difficult, since alcohol is the most popular addictive [29].

So, is the type of alcohol consumed important? A Scottish prospective cohort study including 5766 men aged 35–64 during a 21-year follow-up, after considering the effects of classic risk factors, showed no association between high-percentage alcohol consumption and reduced ischemic mortality. The risk of death from all causes was greatest for people consuming over 22 units of alcohol per week. The study also showed a doubling of the risk of death from stroke in a group of participants consuming over 35 units of alcohol per week. It should be emphasized that in the analyzed population the main sources of ethanol were whiskey and gin, in contrast to the population of France and southern Europe, where red wine occupies the dominant position among alcoholic beverages and in which the lowest cardiovascular mortality in Europe is observed [30,31].

Interpreting the results of observational studies, it should be borne in mind that it is impossible to define a clear cause and effect relationship between alcohol consumption and reduction in cardiovascular morbidity and mortality. Observational studies are mainly based on data from questionnaires, through which participants independently assess the amount or frequency of alcoholic beverages consumed [32]. This may be associated with an underestimation of consumption in the group of people who actually abuse alcohol. In addition, abstainers, who in most epidemiological studies appear to be at greater risk of coronary incidents and death from cardiovascular causes than those who consume moderate amounts of alcohol, are also a group of non-drinkers based on the disease history.

## 3. Holiday Heart Syndrome—Alcohol and Atrial Fibrillation

In 1987, Ettinger et al. designed the study to assess the impact of weekend and holiday alcohol consumption on the risk of atrial fibrillation (AF) and other arrythmias—the phenomenon currently known as Holiday Heart Syndrome (HHS). Patients with HHS are asymptomatic (without ischemic heart disease or heart failure symptoms) and usually have normal laboratory test results [33]. In 1984, Thorton et al. revised the accepted notion that HHS can only occur in chronic alcohol consumers. They presented a couple of cases with acute AF associated with sporadic large amounts of alcohol consumption. In all patients, spontaneous cardioversion was observed within 24 h [34]. A characteristic feature of HHS is the absence of new arrhythmic episodes during abstinence and recurrence after alcohol consumption [35]. In Samokhvalov et al. meta-analysis, increased risk of AF was observed in females consuming >24 g pure ethanol daily and in males ingesting more than 36 g. Lower amounts of alcohol did not raise the risk of arrhythmia [36]. In Gallagher et al. meta-analysis, 5–10% participants consuming excessive amounts of alcohol experienced AF, whereas low levels of ethanol consumption did not significantly influence the frequency of AF [37]. Klein et al. proved that an ethanol concentration of 2% and higher inhibits cardiac sodium channels leading to a sodium—calcium exchanger stimulation that, in turn, prolongs action potential together with a repolarization period, resulting in the increased risk of arrhythmia [38]. Cardy et al. investigated 13 individuals aged 23–27 years old, consuming 0.95 g of ethanol per body mass kilogram. P wave and QRS complex widening reflecting conduction disturbances were observed in every study participant [39]. T Mäki et al. showed an increase in beta-adrenoceptors density in lymphocytes during the alcohol drinking period in patients with a history of alcohol-associated AF, but no change occurred in the density in control subjects (age-matched controls without history of alcohol-associated AF). The observed increased density of beta-adrenoreceptors might be associated with an augmented reaction to adrenergic stimuli. The study also revealed a significantly higher catecholamines concentration in patients with AF than in the control group [40]. Marcus et al., in the group of 100 participants (consenting patients with paroxysmal AF, mean age, 64 years [SD, 15]; 79% male; 85% white) during a 4-week ECG monitoring, noted that 56 individuals experienced at least one AF episode during alcohol consumption. An AF episode was associated with twofold higher odds of one alcoholic drink (odds ratio [OR], 2.02 [95% CI, 1.38 to 3.17]) and greater than threefold higher odds of at least two drinks (OR, 3.58 [CI, 1.63 to 7.89]) in the preceding 4 h. Episodes of AF were also associated with higher odds of peak blood alcohol concentration (OR, 1.38 [CI, 1.04 to 1.83] per 0.1% increase in blood alcohol concentration) [41].

## 4. Deleterious Cardiovascular Effects of Alcohol Abuse—“Binge and Compulsive Drinking”

While it is acknowledged that regular consumption of moderate doses of alcohol can protect against cardiovascular disease, the issue of the effects of alcohol abuse—regular or episodic—has not yet been clearly clarified, especially the phenomenon of “binge drinking”, i.e., drinking large amounts of alcohol in the short term, e.g., drinking ≥ five drinks within one day of the week, with abstinence on the following days. People who admit to this “way of drinking” experience the adverse effects of such alcohol consumption [14]. Based on nearly 8 years of observation, Murray et al. found that occasional consumption of large amounts of alcoholic beverages increases the risk of coronary heart disease in men and women and hypertension in men. On the other hand, regular drinking of small doses of alcohol had a statistically significant cardioprotective effect in both sexes [32]. The theory of the adverse effects of irregular consumption of relatively large amounts of alcohol was also confirmed by studies by Mukamal et al., indicating a twofold increase in the risk of death in people after MI who reported episodes of consumption of three or more “drinks” in a short period of 1–2 h [42]. Nearly 10 years of observation of almost 10,000 men without coronary artery disease at the beginning of the research in the prospective PRIME study showed that binge drinking increases the risk of coronary heart disease and hypertension, while regular consumption of low doses of alcohol had a statistically significant cardioprotective effect in both sexes [43]. In the review by Piano et al., the data from human experimental, prospective cross-sectional and cohort epidemiological studies show that binge drinking is associated with a higher risk of pre-hypertension, hypertension, MI and stroke in middle-aged and older adults. Moreover, it may have adverse cardiovascular effects in young adults aged 18 to 30. It is suggested that binge drinking may cause oxidative stress, vascular injury and be proatherogenic [44].

The results of prospective studies have largely confirmed the results of clinical studies. Alcohol abusers have a higher risk of mortality from all causes [45]. It was observed that people who had sudden cardiac death more often consumed alcohol in the last hours of life, compared with patients who died of other cardiovascular diseases [46]. This regularity was confirmed by the results of the British Regional Heart Study, which showed that the incidence of sudden cardiac death in the group of alcohol abusers was about twice as high as among persons who did not abuse alcohol. The increase in the incidence of sudden deaths was most pronounced in older men, even if coronary artery disease was not diagnosed [47].

The relationship between compulsive drinking, i.e., a strong irrational need, even hunger for drinking, and cardiovascular diseases is expected to be systematically analyzed. Compulsive drinkers are thought to be at an increased risk of sudden cardiac death (most commonly in the mechanism of ventricular arrhythmia) and the development of cardiomyopathy. There are also data suggesting that compulsive drinking may increase the risk of MI. It is believed that the dominant drinking pattern—compulsive drinking—may underlie the dramatic increase in mortality from cardiovascular disease in the former Soviet Union, and currently in the Russian Federation [48]. In some regions (e.g., some areas in Russia and Eastern Europe), even every second death among men before 54 is due to alcohol addiction and abuse [48,49]. A study conducted in the United States showed that mortality was almost twice as high in people drinking small amounts of alcohol, who had compulsive drinking episodes than in those drinking the same amounts of alcohol without compulsive episodes [45]. A causal connection between the pattern of alcohol consumption and adverse epidemiological phenomena was also confirmed by other types of observation. Both in Russia in 1985 and Poland in the years 1981–1982, a decline in death rate from cardiovascular causes was observed. In both countries, these periods coincided with the reduced availability of high-percentage alcohol in Russia resulting from the anti-alcohol policy of Mikhail Gorbachev under the so-called perestroika, and in Poland, it was associated with the limited distribution of alcohol during martial law [49,50].

It has been shown that the effect of alcohol on mortality varies depending on the age group. In younger age groups (under 50 years of age), the effects of alcohol are clearly unfavorable mainly due to the increased risk of death from injuries and accidents, especially in the group of people aged 15–29. On the other hand, among the elderly, the number of deaths, mainly due to cardiovascular diseases, which could be prevented by moderate alcohol consumption, is higher than the number of deaths caused by drinking [51]. It should be noted that most studies conducted to date on the relationship between alcohol consumption and risk of death included middle-aged and older people, excluding young people. Studies that also included the younger population indicate that there is no protective effect associated with moderate alcohol consumption in young adults [52]. This is probably due to the fact that in older age groups, where cardiovascular diseases are the predominant cause of death, the benefits of drinking alcohol are more pronounced than in people in the age group of 15–29 who more often die from injuries and accidents, and alcohol may even increase the risk of death from these causes [53].

It should also be borne in mind that the conclusion on the protective effect of small doses of alcohol could also result from a sample selection error because people with health problems could have stopped drinking, which would artificially increase mortality in the non-drinkers group [54]. However, the available data do not support this hypothesis. In several studies, among non-alcohol drinkers, there were identified individuals who stopped drinking for various reasons and abstainers. Compared to moderate alcohol drinkers, both abstainers and former drinkers had a higher risk of dying from cardiovascular disease, while no significant differences were found between these groups [52]. Moreover, the results of most studies did not change after excluding from the analysis, deaths that occurred during the first years of observation (it was assumed that these deaths could have been a consequence of previous diseases) [55] (Table 1).

## 5. Are Moderate Alcohol Consumers Simply Living a Healthier Life?

Some authors suggest that regular and moderate alcohol consumption while maintaining a healthy lifestyle (physical activity, a proper diet with large vegetable intake and no addictions) can significantly reduce the risk of cardiovascular disease and death, especially heart attack, and even prolong life expectancy [58,59,60,61]. On the contrary, some recent analyses indicate that association of low-to-moderate alcohol consumption with the reduction in cardiovascular risk is a result of lifestyle changes and that any reduction in alcohol consumption is in fact beneficial in terms of general health [56,57].

Biddinger et al. conducted an observational study in the group of 371,463 participants to assess the relationship between habitual alcohol consumption and the risk of cardiovascular diseases (hypertension, CAD, MI, stroke, HF and AF). Simultaneously, authors also aimed to evaluate the influence of six other lifestyle factors (smoking frequency, normalized BMI, self-reported physical activity, cooked vegetable intake, red meat consumption and self-reported health). Drinking groups were defined as abstainers (0 drinks/week), light (>0–8.4 drinks/week), moderate (>8.4–15.4 drinks/week), heavy (>15.4–24.5 drinks/week) and abusive (>24.5 drinks/week). [A standard drink is any drink that contains about 14 g of pure alcohol.] Alcohol intake comprised 38% beer, 29% red wine, 24% champagne or white wine, 6% spirits, 3% fortified wine and 0.2% other alcoholic beverages. Investigators found that light-to-moderate drinkers had the lowest heart disease risk, followed by people who abstained from drinking. People who drank heavily had the highest risk. They also found that light-to-moderate drinkers tended to have healthier lifestyles than abstainers; moreover, taking just a few lifestyle factors into account significantly lowered any benefit associated with alcohol consumption. The genetic analyses also revealed differences in cardiovascular risk across the spectrum of alcohol consumption, with minimal increases in risk when going from zero to seven drinks/week, much higher risk increases when progressing from seven to fourteen drinks/week (in both men and women) and very high risk—twenty-one or more drinks/week. Authors suggest a rise in cardiovascular risk even at levels deemed low-risk—below two drinks per day for men and one drink per day for women [56].

Hu et al., in the group of 40,386 males, proved the harmful effect of alcohol consumption on both cardiovascular and all-cause mortality, underlining potential health benefits associated with the reduction in alcohol intake even in the group of low-to-moderate consumption [57].

Evidences from different experimental studies have suggested that these beneficial effects are due to polyphenols found in red wine, especially resveratrol. Therefore, there is a need for further clinical studies, especially randomized, double-blind, placebo-controlled trials, to objectively confirm the possible health-promoting effects of this substance and to determine both the efficacy and safety and possible therapeutic potential [62,63]. Moreover, resveratrol can potentially increase plasma concentration of direct oral anticoagulants such as dabigatran, edoxaban and betrixaban [64].

On the contrary, obesity, poor diet, low physical activity, smoking and excessive alcohol consumption are correlated with a higher incidence of CVD, dementia and diabetes [65,66]. 

## 6. Conclusions

The possibility of the protective effect of small and moderate doses of alcohol on cardiovascular diseases is significant from the point of view of public health. All over the world, cardiovascular diseases are the main cause of death, so even a minimal reduction in their relative risk can translate in absolute terms into a significantly lower number of incidence and death from cardiovascular diseases. However, due to the fact that alcohol consumption is associated with many health hazards, it is not recommended to consume it as a preventive action of cardiovascular diseases [67].

## Figures and Tables

**Table 1:** Summary of selected studies.

Authors/Year	Design/Number of Participants	The Most Important Outcome
Millwood et al. 2019 [5]	mendelian randomization, prospective study/512,715	Protective effects of moderate alcohol intake against stroke are non-causative. Alcohol consumption uniformly increases blood pressure and stroke risk.
Holmes et al. 2014 [6]	mendelian randomization, meta-analysis of 56 studies/261,991	Individuals with a genetic variant associated with non-drinking and lower alcohol consumption had a more favourable cardiovascular profile and a reduced risk of coronary heart disease than those without the genetic variant. Reduction in alcohol consumption is beneficial for cardiovascular health.
Doll et al. 1994 [9]	prospective study/ 12,321	The consumption of alcohol appeared to reduce the risk of ischemic heart disease, largely irrespective of amount. Among regular drinkers, mortality from all causes combined increased progressively with amount drunk above 168 g of pure alcohol a week. Among British men in middle or older age, the consumption of an average of one or two units of alcohol a day is associated with significantly lower all-cause mortality vs. non-drinkers.
Hoffmeister et al. 1999 [10]	prospective study/ 17,770	Higher serum HDL cholesterol levels were observed with increasing alcohol intake. Men who consumed 1–20 g alcohol/day had a significantly lower all-cause and cardiovascular mortality vs. non-drinkers.
Ronksley et al. 2011 [14]	meta-analysis of 84 studies	Dose-response analysis revealed that the lowest risk of coronary heart disease mortality occurred with one to two drinks a day. Secondary analysis of mortality from all causes showed lower risk for drinkers compared with non-drinkers.
Mukamal et al. 2010 [16]	retrospective study/245,207	Light and moderate volumes of alcohol consumption were inversely associated with cardiovascular mortality.
Biddinger et al. 2022 [56]	prospective study/371,463	Genetic epidemiology suggested that alcohol consumption of all amounts was associated with increased cardiovascular risk, but marked risk differences exist across levels of intake, including those accepted by current national guidelines.
Hu et al. 2022 [57]	prospective study/ 40,386	The risk of incident CVD and all-cause mortality was increased by 27% and 20% per standard drink increment of genetically predicted alcohol consumption, respectively. The authors show the potential health benefits of lowering alcohol consumption, even among light-to-moderate male drinkers.

## Data Availability

No new data were created or analyzed in this study. Data sharing is not applicable to this article.
